# Plausibility or Honesty?

**Published:** 1890-05-17

**Authors:** 


### Editor's Letter-Box.

[Our Correspondents are reminded that prolixity is a great bar to publication, and that brevity of style and conciseness of statement greatly facilitate early insertion.]

### PLAUSIBILITY OR HONESTY?

Sir,—I think some protestation should be made against the remarks made in your issue of May 3rd as regards "women's chronic diseases." I refer to the following:—"We cannot but affirm that ladies are willing victims to unworthy medical arts; plausibility in men is the god of their idolatry; and so long as a doctor can please their minds he may do what he will with their husbands' purses." As a woman, I must say it is perfectly unfair to attribute such unworthy motives to a woman's preference for a doctor. No woman possessed of right principle or common sense would prefer a doctor on account of his "plausibility"; she could only be guided in her opinion by his capability. The writer of the quoted annotation, whoever he may be, may have had an unfortunate experience of women and their consciences; but it is absolutely unjust to attribute, as he does, such wholesale carelessness of "their husbands' purses" to the sex generally. The writer asks if women want "plausibility or honesty"? I think the right answer to that question is that women possessed of sense and principle expect, and should obtain, from every doctor honesty, skill, and courtesy.—I am, Sir, One who Loves Justice.

May 7th, 1890.

